# Supplementary material for: Animating hydrogel knotbots with topology-invoked self-regulation
Source: Nat Commun. 2024 Jan 5;15:300. doi: 10.1038/s41467-023-44608-x (PMC10770334; doi:10.1038/s41467-023-44608-x)
Supplement: Supplementary file 1 — Supplementary Information [file 41467_2023_44608_MOESM1_ESM.pdf]

Supplementary Information for

Animating hydrogel knotbots with topology-invoked self-regulation

Qing Li Zhu,[1]† Weixuan Liu,[2]† Olena Khoruzhenko,[3] Josef Breu,[3] Wei Hong,[2]* Qiang Zheng,[1]* Zi Liang Wu[1]*

[1] Ministry of Education Key Laboratory of Macromolecular Synthesis and Functionalization, Department of Polymer Science and Engineering, Zhejiang University, Hangzhou 310058, China;

[2] Shenzhen Key Laboratory of Soft Mechanics & Smart Manufacturing, Department of Mechanics and Aerospace Engineering, Southern University of Science and Technology, Shenzhen 518055, China;

[3] Bavarian Polymer Institute and Department of Chemistry, University of Bayreuth, Universitätsstrasse 30, Bayreuth 95440, Germany.

†They contributed equally to this work.

*Corresponding authors. E-mail: wuziliang@zju.edu.cn (Z.L.W.), zhengqiang@zju.edu.cn (Z.Q.), hongw@sustech.edu.cn (W.H.)

This Supplementary Information includes:

Supplementary Methods

Supplementary Discussions

Supplementary Figures

Supplementary Table

Supplementary References

Supplementary Methods

Materials. *N*-isopropylacrylamide (NIPAm) was received from Aladdin Chemistry Co., Ltd. and used as the monomer. *N*,*N'*-methylenebis(acrylamide) (MBAA) was purchased from Sigma Aldrich and used as the chemical crosslinker. Ethyl phenyl (2,4,6-trimethylbenzoyl) phosphinate and lithium bromide were received from Aladdin Chemistry Co., Ltd. Chloride solution ($AuCl_4 \cdot 3H_2O$) and sodium citrate dihydrate were purchased from Sigma Aldrich. 2-Butanone solution was purchased form Sinopharm Chemical Reagent Co., Ltd. Lithium phenyl-2,4,6-trimethylbenzoylphosphinate (LAP) was synthesized and used as the photo-initiator[1]; the synthesis details of LAP is described in the following section. Fluorohectorite $[Na_{0.5}][Li_{0.5}Mg_{2.5}][Si_4]O_{10}F_2$ nanosheets (NSs) were obtained by melt synthesis according to the procedure reported in the literature[2], which can be scalable up to several tens of grams per batch. The NS powders spontaneously swelled and delaminated into single lamellae with the thickness of 1 nm, when immersed in deionized water. The NSs had a high charge density of 1.1 nm^{-2}, a large aspect ratio of ~20,000, and anisotropic mechanical properties. They were stiff in the in-plane direction with Young's modulus of 150 GPa and flexible in the cross-plane direction[3]. The aqueous suspensions of NSs with the content of 0.5 wt%, 1.0 wt%, and 2.0 wt% showed a nematic phase and high stability at room temperature. Gold nanoparticles (AuNPs), which were widely used as high-efficiency photothermal agents, were synthesized in a large quantity according to the citrate reduction method[4]. The average size of synthesized AuNPs was 9.5 ± 0.7 nm, as characterized by TEM. The AuNPs and the NSs had a high stability after being incorporated into the hydrogel, which afforded the nanocomposite gel with photo-response and anisotropic deformation.

Synthesis of LAP. 12.6 g of ethyl (2,4,6-trimethylbenzoyl) phenylphosphinate and 13.9 g of lithium bromide were dissolved in 227 mL of 2-butoanone at room temperature[1]. The obtained solution was heated to 65 °C for 40 min, and some white precipitates were formed. The reaction mixture was cooled to room temperature to obtain more white precipitates. The precipitates were filtered to obtain crude LAP, which was washed using 2-butanone to remove the unreacted lithium bromide. The LAP powders were dried in a vacuum oven of 70 °C for 3 days and stored in dark before use.

Synthesis of AuNPs. 4.43 mL of chloroauric acid was added to 192 mL water to form a seed solution in a flask. Then, the seed solution was stirred with a speed of 420 rpm and heated to the boiling point by using an oil bath. 4 mL of aqueous solution of sodium citrate dihydrate (concentration, 20 mg mL^{-1}) was added to the seed solution with continuous heating and stirring for another 15 min. After the reduction reaction, the mixture became wine red color and cooled to room temperature. The aqueous suspension of AuNPs was concentrated by centrifuge at 13,000 rpm and stored in the fridge of 4 °C before use.

Characterizations

Birefringence measurement. The birefringence of the anisotropic cylindric hydrogels was observed under a polarizing optical microscope (LV100N POL, Nikon) with and without 530 nm tint plate. The photos were recorded by a CCD camera. The light intensity of photos of hydrogels under different shear rate was evaluated using ImageJ software. The light intensity ratio (I/I_0) is calculated to show orientation of NSs, where I and I_0 are the transmitted light intensity of the sample (distance to edge, 0.2 mm) under crossed polarizers and parallel polarizers, respectively[5,6].

SAXS measurement. The alignment of NSs was characterized by SAXS measurements on BL 16B1 beamline at Shanghai Synchrotron Radiation Facility (SSRF). X-ray wavelength was 0.124 nm, the sample-to-detector distance was 1954 mm and the data were collected via Pilatus 2M detector (DECTRIS, Switzerland) with a resolution of 1475 × 1679 pixels. The 2D SAXS patterns was transformed into 1D profiles [$I(q) \sim q$ plot] and [$I(\varphi) \sim \varphi$ plot] was obtained with the Fit2D software, where I is the scattering intensity, q is scattering vector and φ is the azimuthal angle. $I(\varphi) \sim \varphi$ plots used to calculate the orientation degree (π) according to the classical equation of $\pi = (180–H)/180$, where H is the half width of the peak of the azimuthal plot from the selected equatorial reflection.

Viscosity Measurement. The viscosity of the suspensions with different content of NSs was measured using a DHR-2 rheometer (TA Instruments). The test used a cone-plate geometry with diameter of 40 mm and angle of 1.995°. The measurements were conducted at 25°C and the shear rates in the rage from 0.001 to 1000 s^{-1}.

SEM observation. Microstructures of cylindrical PNIPAm gel without NSs and anisotropic

nanocomposite PNIPAm gel were observed with a field-emission scanning electron microscope (SEM; Hitachi S4800). The samples were prepared by drying at room temperature and then fractured in liquid nitrogen. The fractured cross-section of the gel was coated with a thin layer of gold using a sputter coater.

TEM observation. The orientation structure of NSs were observed with transmission electron microscope (TEM; HT-7700) at an acceleration voltage of 120 kV, the sample is dried and sliced at room temperature.

Mechanical test. Mechanical properties of the hydrogels were measured on a tensile tester (Instron 3343) at room temperature. The samples were cut into a dumbbell shape with an initial gauge length of 12 mm and a width of 2 mm. The stretch rate was 100 mm min^{-1}. Young's modulus was calculated from the initial slop of the tensile stress-strain curve with a strain below 10%.

Supplementary Discussion

Assumptions and limitations of the models. Although the simulation results qualitatively align with the experimental observations in this study, there are some differences between the computational models and the real situation. It is crucial to highlight that these differences can largely be attributed to simplifying assumptions made for the sake of computational tractability. We ensure that the models align with the experimental results through the following assumptions. (i) In the experimental setup, it is observed that the light intensity decays non-linearly within the gel. However, as the detailed distribution of light intensity is unknown and not deemed as an important factor as suggested by our numerical tests, a heat source field that decays linearly with depth is used for simplicity in the simulations. (ii) In reality, the friction of the gel against the substrate and that between two parts of a gel should both be functions of the relative velocity. To facilitate computation while retaining a reasonable level of fidelity, we employ constant friction coefficients for both cases. These approximations and simplifications have proven effective in ensuring a close match with the experimental results.

While reasonable assumptions make the simulations coincide with the experimental results, it is also important to acknowledge certain limitations in specific situations. (i) In the cases of significant reflection or refraction of light, a pre-defined temperature field may be inadequate. A detailed model of the light intensity distribution can be incorporated for more accurate representation. (ii) If thermal conduction is significant, our simple treatment of taking thermal radiation as the only means of heat dissipation may be less accurate, and a more complete model including heat conduction may be needed. The extensions are deemed as unnecessary in the current study, and will be an interesting subject for future study.

Supplementary Figures

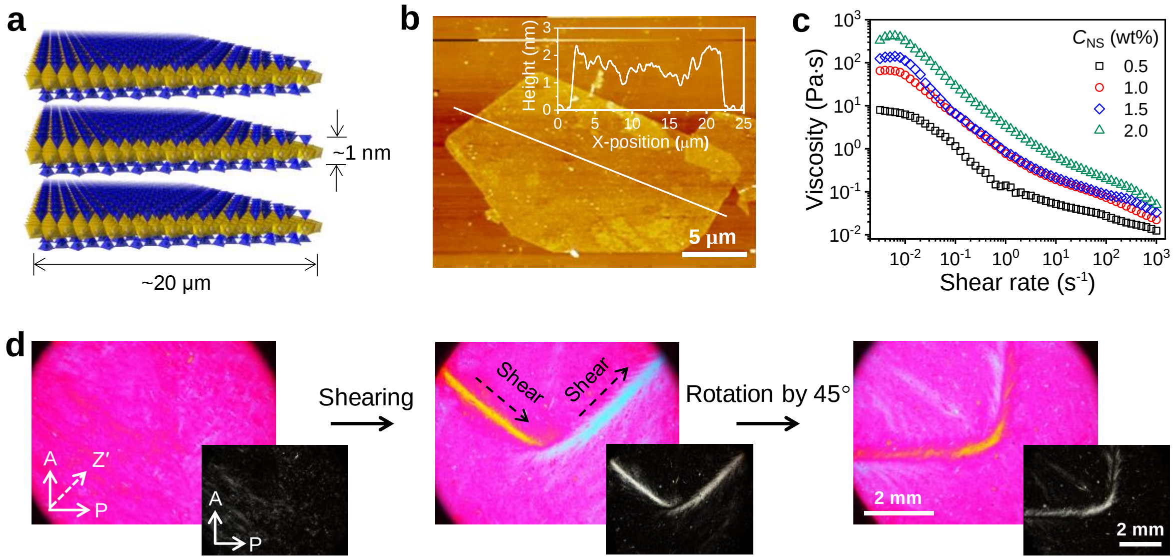

Supplementary Figure 1. Structure and properties of $[Na_{0.5}][Li_{0.5}Mg_{2.5}][Si_4]O_{10}F_2$ nanosheets and their aqueous suspensions. a Schematic structure of the fluorohectorite nanosheets. **b** Topographical atomic force microscope (AFM) image of the nanosheet. **c** Viscosity of aqueous suspensions of NSs at different concentrations (C_{NS}) as functions of the shear rate at room temperature. Representative images of n = 3. **d** POM images of an aqueous suspension of NSs (C_{NS} of 1.0 wt%) that are aligned by a mechanical shear. A: analyzer; P: polarizer; Z′: slow axis of the 530 nm tint plate. Representative images of n = 3.

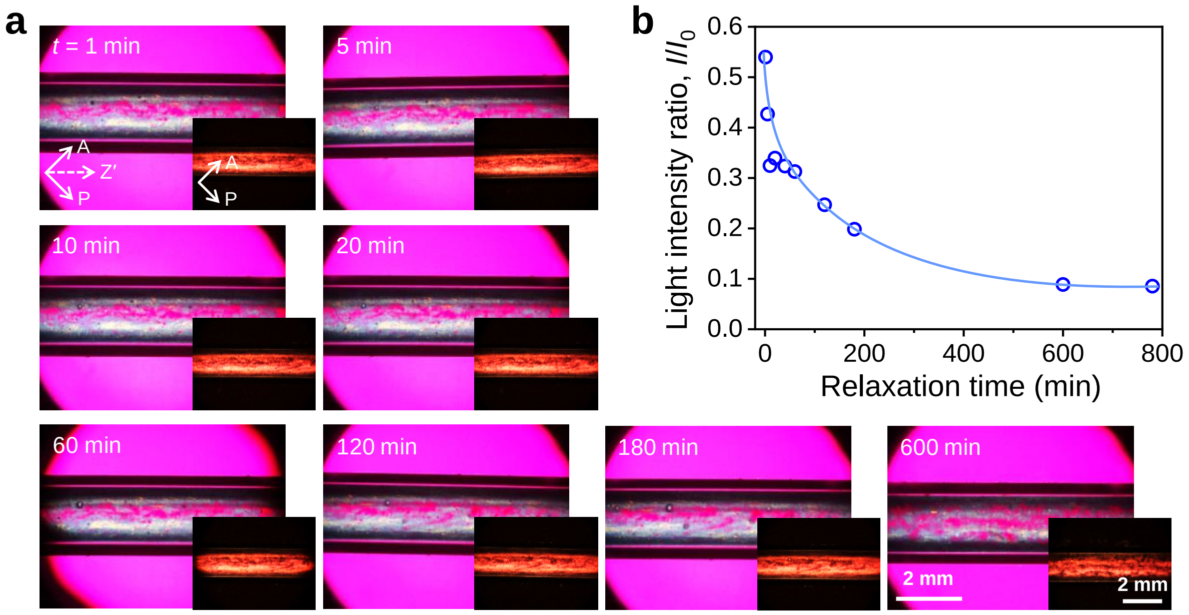

Supplementary Figure 2. Relaxation behavior of the aligned NSs in a suspension after shearing. a POM images taken at different time after shearing at room temperature. Representative images of n = 3. **b** Variation of the transmitted light intensity ratio (I/I_0) of the sample through crossed and parallel polarizers during the relaxation process. I and I_0 are the transmitted light intensity of the sample (distance to edge, 0.2 mm) through crossed polarizers and parallel polarizers, respectively. C_{NS}, 1.0 wt%; flow rate, 10.6 mm s^{-1}. A: analyzer; P: polarizer; Z′: slow axis of the tint plate.

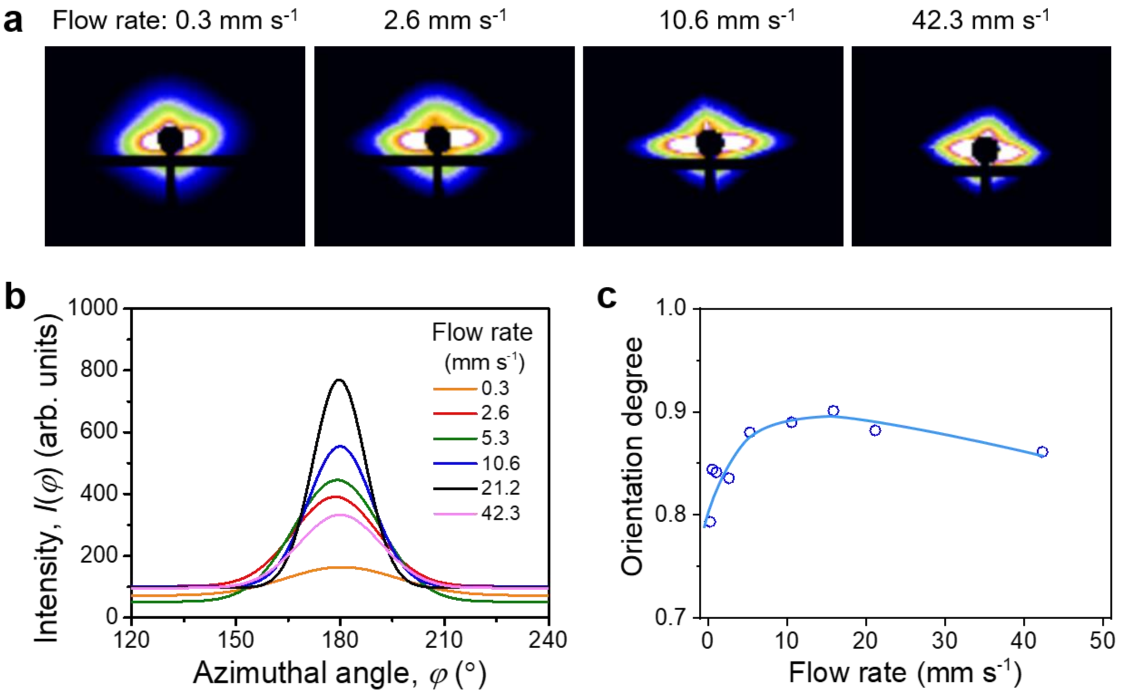

Supplementary Figure 3. SAXS measurement of the nanocomposite hydrogels prepared with different flow rate to align the NSs. a 2D SAXS patterns of the cylinder hydrogels. **b** Corresponding intensity-azimuthal angle curves. **c** Calculated orientation degree of the NSs within the hydrogel as a function of flow rate. The incident X-ray beam is irradiated at the position with a distance of 0.2 mm from the edge of the cylindrical gel. C_{NS} is 1.0 wt%. The orientation degree of NSs within the hydrogel increases from 0.8 to 0.9 with the increase in flow rate from 0.3 to 21.2 mm s^{-1}. However, further increase in the flow rate leads to slight decrease in the orientation degree of NSs, because the suddenly ceasing of the flow after a quick injection may destroy the alignment of NSs to some extent before the polymerization and gelation process.

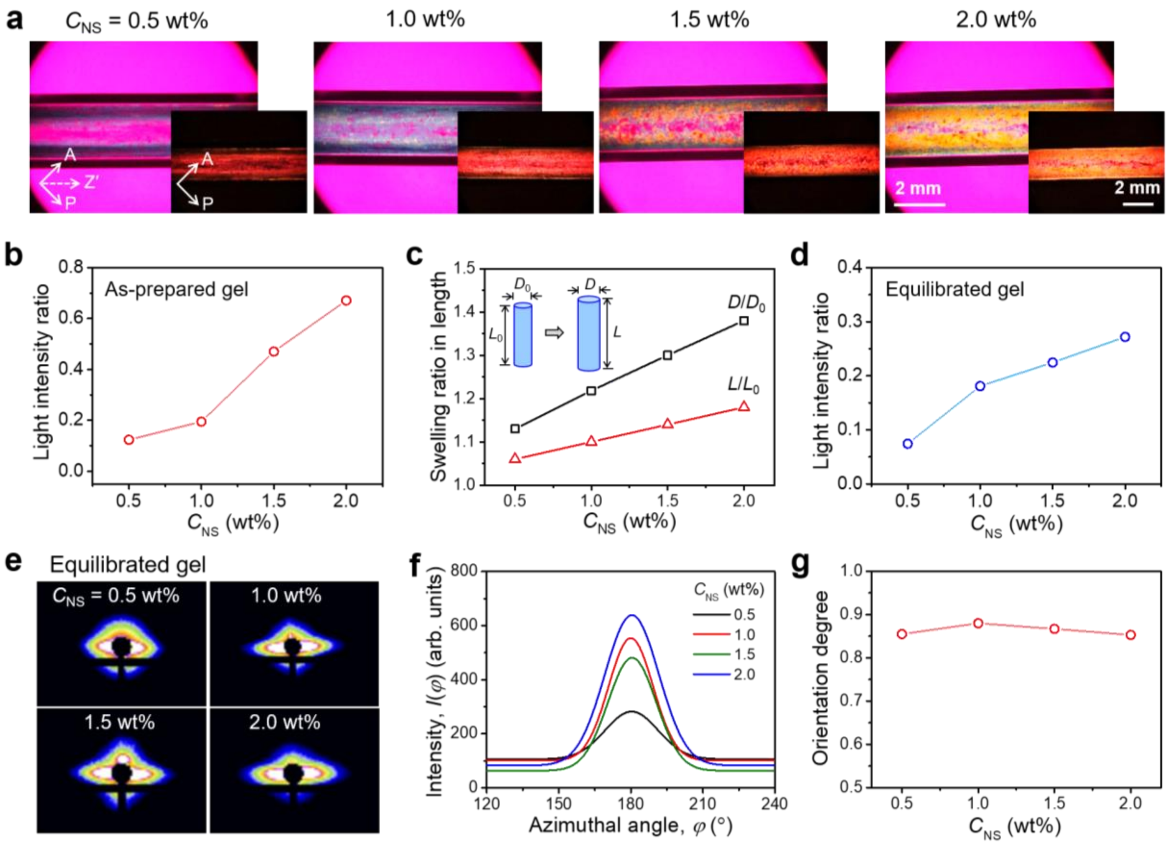

Supplementary Figure 4. Effect of C_{NS} on the anisotropic properties of the nanocomposite hydrogels. a POM images of the anisotropic hydrogels with different C_{NS}, which are prepared by shearing and polymerization of the precursor suspensions. Flow rate, 10.6 mm s^{-1}. Representative images of n = 3. **b** Birefringence ratio of the as-prepared hydrogels as a function of C_{NS}. Birefringence intensity ratio is defined as I/I_0, where I and I_0 are the transmitted light intensity of the sample (distance to the edge, 0.2 mm) under crossed polarizers and parallel polarizers, respectively. **c** Anisotropic swelling behavior of the cylindrical gels with different C_{NS}. **d** Birefringence ratio of the equilibrated hydrogels as a function of C_{NS}. **e-g** 2D SAXS patterns (e), corresponding intensity-azimuthal angle curves (f), and orientation degree of NSs (g) within the equilibrated hydrogels of different C_{NS}. The incident X-ray beam is irradiated at the position with a distance of 0.2 mm to the edge of the cylindrical gel.

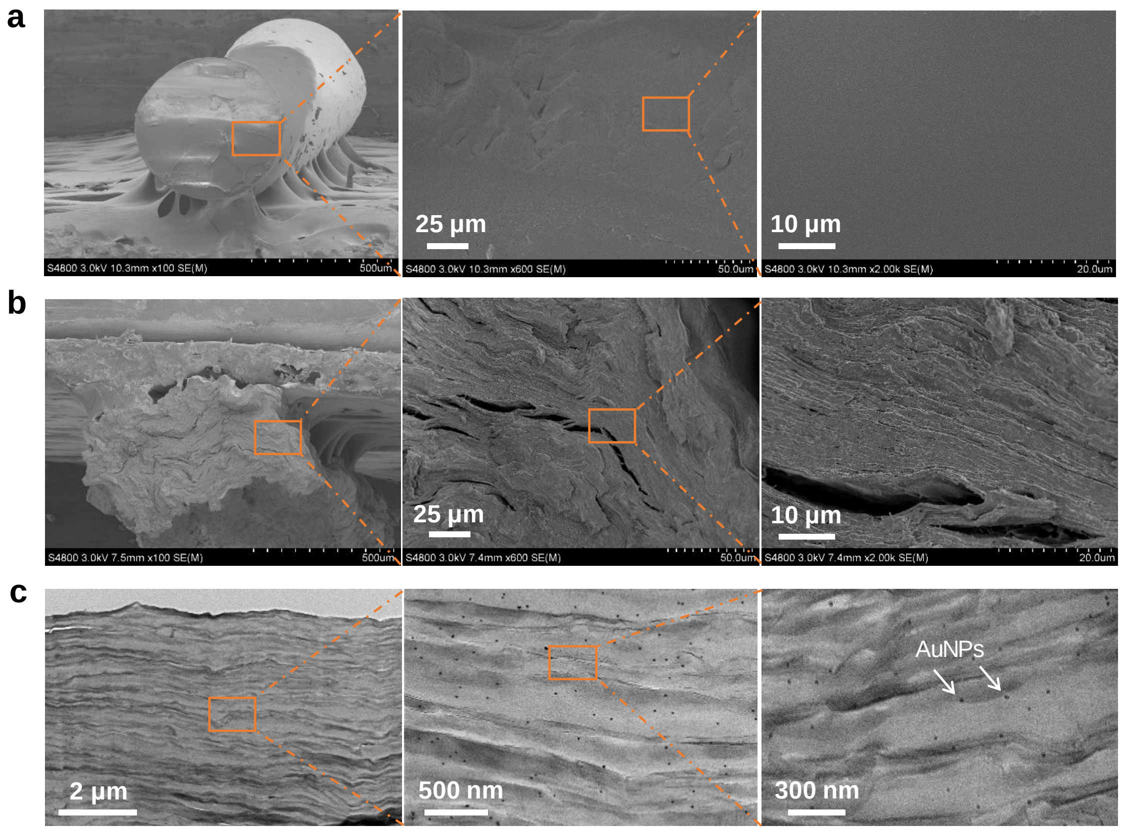

Supplementary Figure 5. SEM and TEM images of the cylindrical hydrogels. a Cross-section SEM images of the PNIPAm hydrogel without NSs. **b** Cross-section SEM images of the anisotropic cylindrical hydrogel. **c** TEM images of the anisotropic cylindrical hydrogel. The nanocomposite hydrogel with C_{NS} of 1.0 wt% and C_{AuNP} of 0.32 wt% is prepared after injection of the precursor suspension with a flow rate of 10.6 mm s^{-1} for the orientation of NSs at room temperature. Representative images of n = 3.

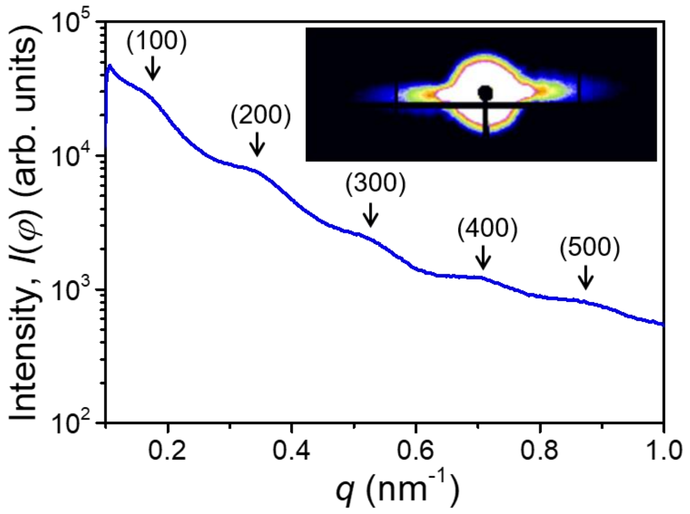

Supplementary Figure 6. SAXS measurement of the anisotropic nanocomposite hydrogel. a 1D SAXS profile of the anisotropic cylinder gel in the as-prepared state at room temperature. $I(q)$ is the scattering intensity, and q is the scattering vector. The cylinder gel is prepared with C_{NS} of 1.0 wt% and flow rate of 10.6 mm s^{-1}.

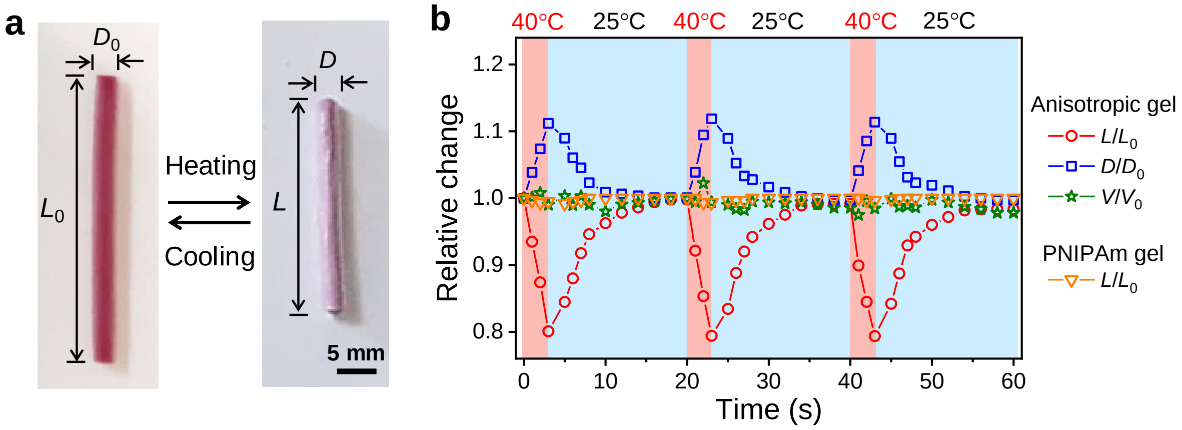

Supplementary Figure 7. Varying dimensions of the anisotropic nanocomposite hydrogel upon cyclic heating and cooling. a Photos showing the variation of the anisotropic hydrogel's dimensions upon cyclic heating and cooling. **b** Variations in length (L/L_0), diameter (D/D_0), and volume (V/V_0) of the anisotropic gel with oriented NSs and isotropic PNIPAm gel without NSs after switching the incubation bath with different temperature (25 and 40 °C). L and L_0 represent the lengths, D and D_0 the diameters, V and V_0 the volumes of the hydrogel before and after the heating, respectively.

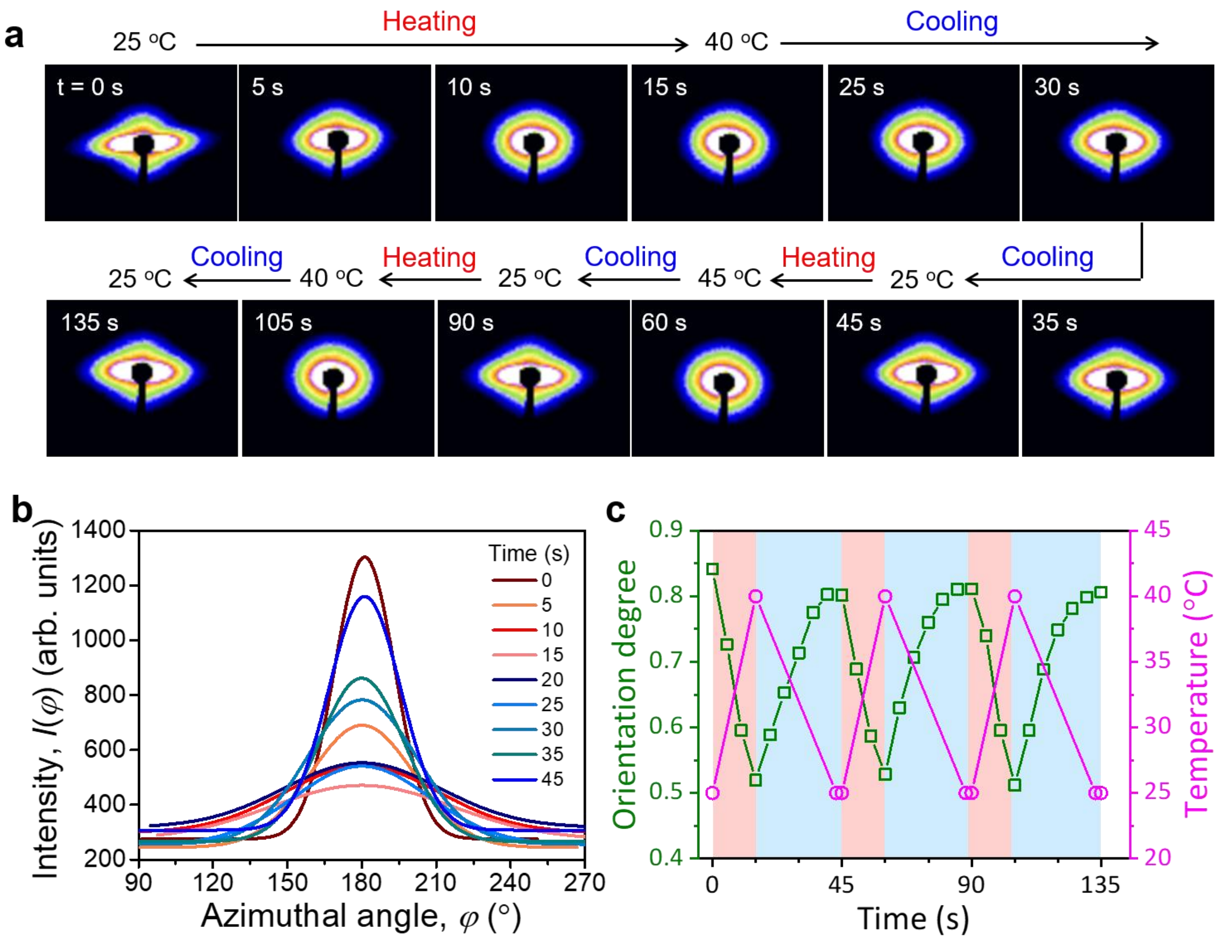

Supplementary Figure 8. Variations of the anisotropic structure of NSs within the nanocomposite hydrogel upon heating and cooling. a 2D SAXS patterns of the anisotropic hydrogel upon heating and cooling between 25 and 40 °C. The heating rate is 60 °C min^{-1}, and the cooling rate is 30 °C min^{-1}. **b** Scattering intensity-azimuthal angle curves of the anisotropic gel during the heating process from 25 to 40 °C with a heating rate of 60 °C min^{-1}. **c** Variation of the orientation degree of NSs within the anisotropic gel during the cyclic heating and cooling process.

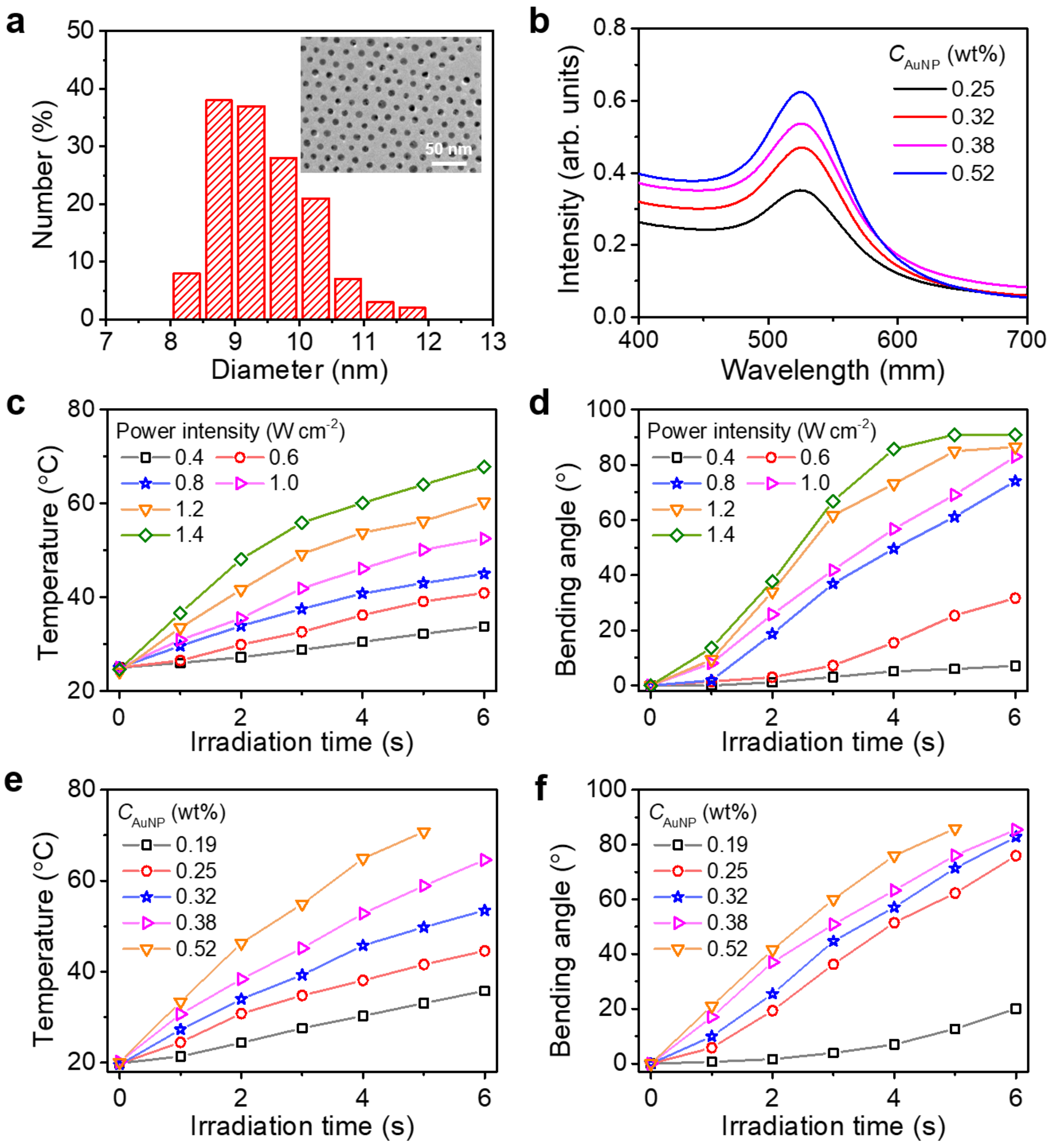

Supplementary Figure 9. Photothermal properties of the anisotropic hydrogels containing different content of AuNPs. **a** TEM image and size distribution of the synthesized AuNPs. Diameter of AuNPs is 9.5 ± 0.7 nm. Representative images of n = 3. **b** Absorption spectra of aqueous suspensions with different content of AuNPs at room temperature. **c**, **d** Variations of temperature (c) and bending angle (d) of the anisotropic gels with C_{AuNP} of 0.32 wt% upon laser irradiation at different power intensities. **e**, **f** Variations of temperature (e) and bending angle (f) of the gels with different C_{AuNP} upon laser irradiation with power intensity of 0.8 W cm^{-2}. The anisotropic gels are synthesized with C_{NS} of 1.0 wt% and diameter of 2.0 mm.

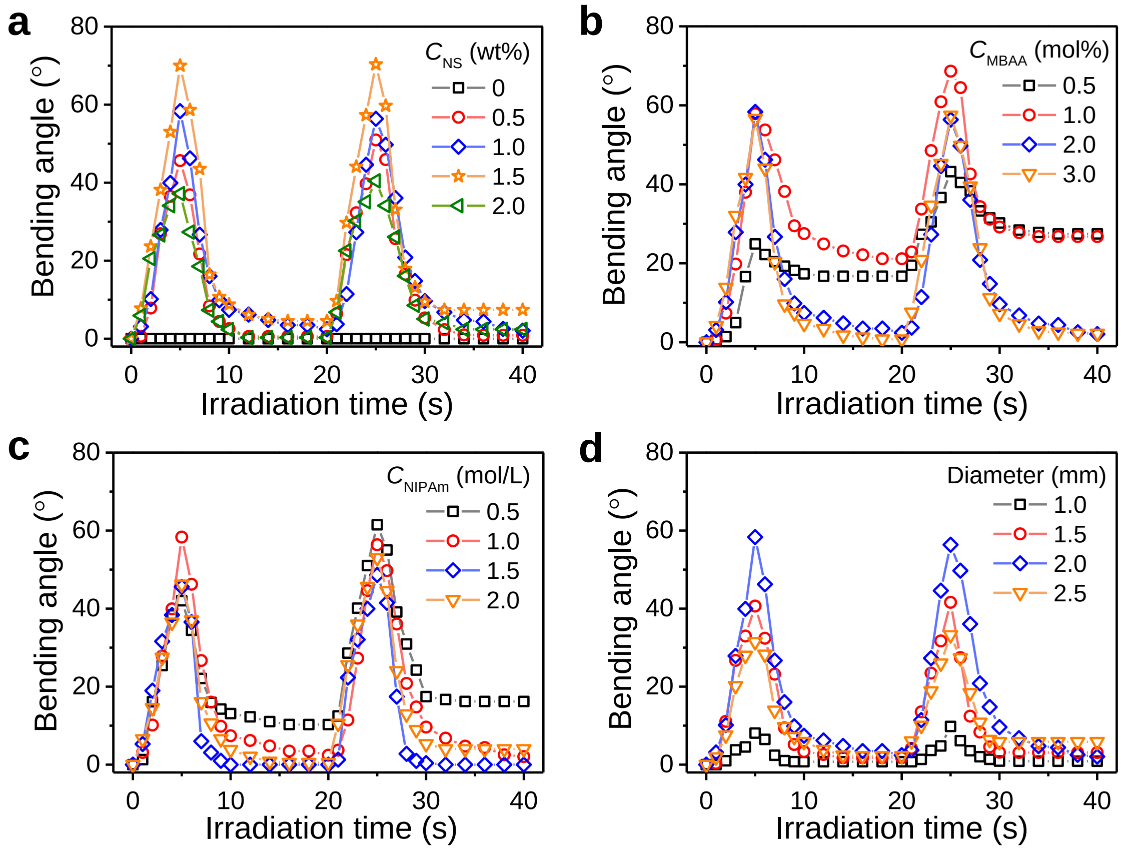

Supplementary Figure 10. Optimization of compositions and diameter of the anisotropic gels by examining the light-induced bending behavior. a-c Effects of component compositions on the bending deformation of the anisotropic gels upon laser irradiation at room temperature: (a) content of NS, C_{NS}; (b) concentration of chemical crosslinker (relative to monomer), C_{MBAA}; (c) concentration of monomer, C_{NIPAm}. **d** Influence of the diameter of the cylinder gels on the light-induced bending behavior at room temperature. The reference compositions and diameter of the anisotropic gels: C_{NS} of 1.0 wt%, C_{MBAA} of 2.0 mol%, C_{NIPAm} of 1.0 mol/L, and diameter of 2.0 mm.

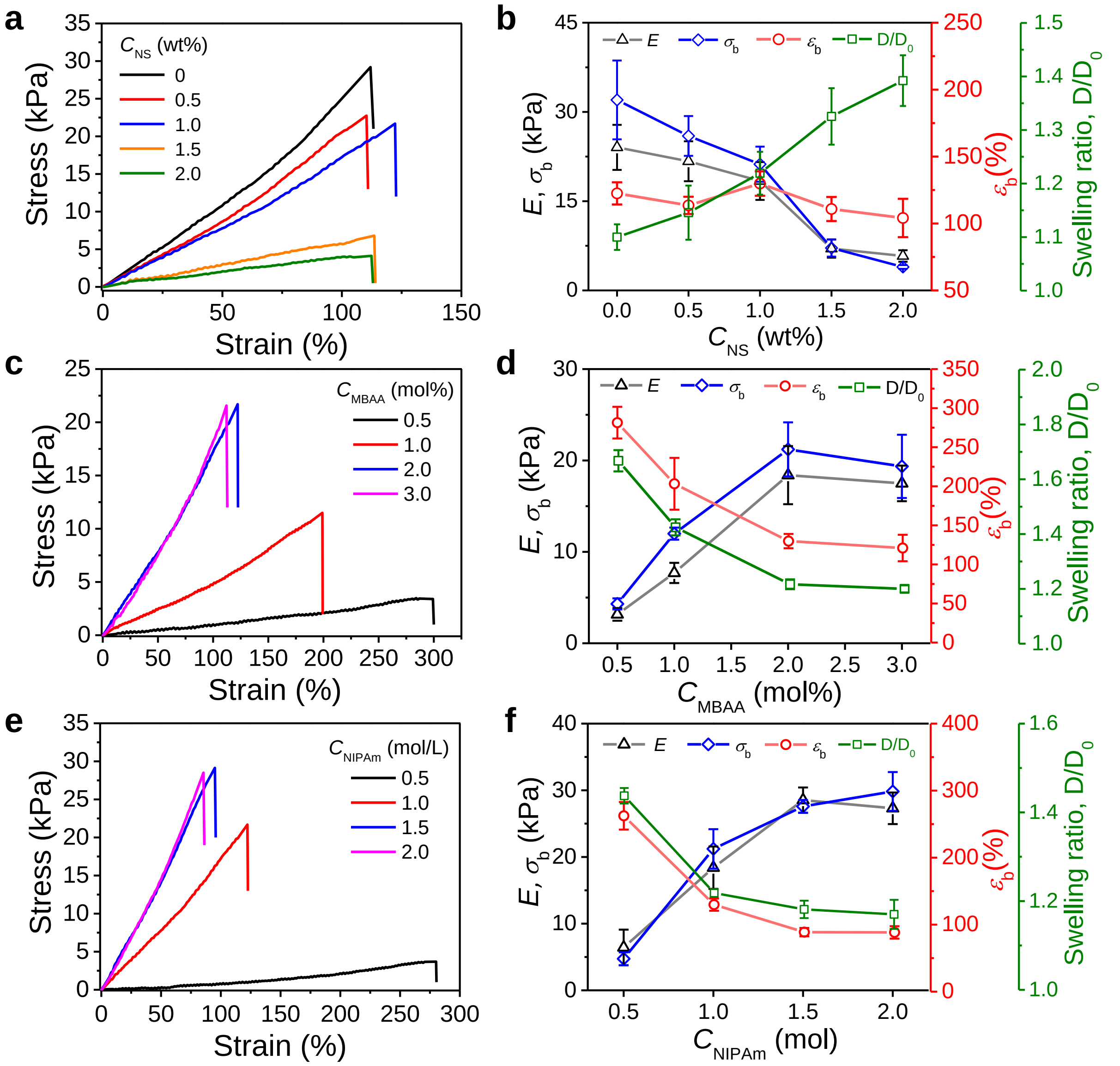

Supplementary Figure 11. Mechanical properties of the anisotropic hydrogels with different compositions. a, **c**, **e** Tensile strain-stress curves of the gels with different content of NS, C_{NS} (a), different concentration of crosslinker, C_{MBAA} (c), and different concentration of monomer, C_{NIPAm} (e). **b**, **d**, **f** Corresponding mechanical parameters of the gels with different C_{NS} (b), different C_{MBAA} (d), and different C_{NIPAm} (f). Three parallel tests are performed to each gel at room temperature; swelling ratio in length of the gel is also presented in the figure. The swelling ratio D/D_0 is examined by measuring the diameter of the cylinder gel before and after the swelling process at room temperature. Anisotropic gel sheets with thickness of 0.6 mm are prepared for the tensile tests. The precursor solutions are subject to reciprocating shear with a rate of 10.6 mm s^{-1} at room temperature and then immediately exposed to UV light for polymerization to fix the anisotropic structure of NSs. The resultant gels are incubated in large amount of water to reach the equilibrium state at room temperature. Data points are represented as mean ± s.d. (n ≥ 3).

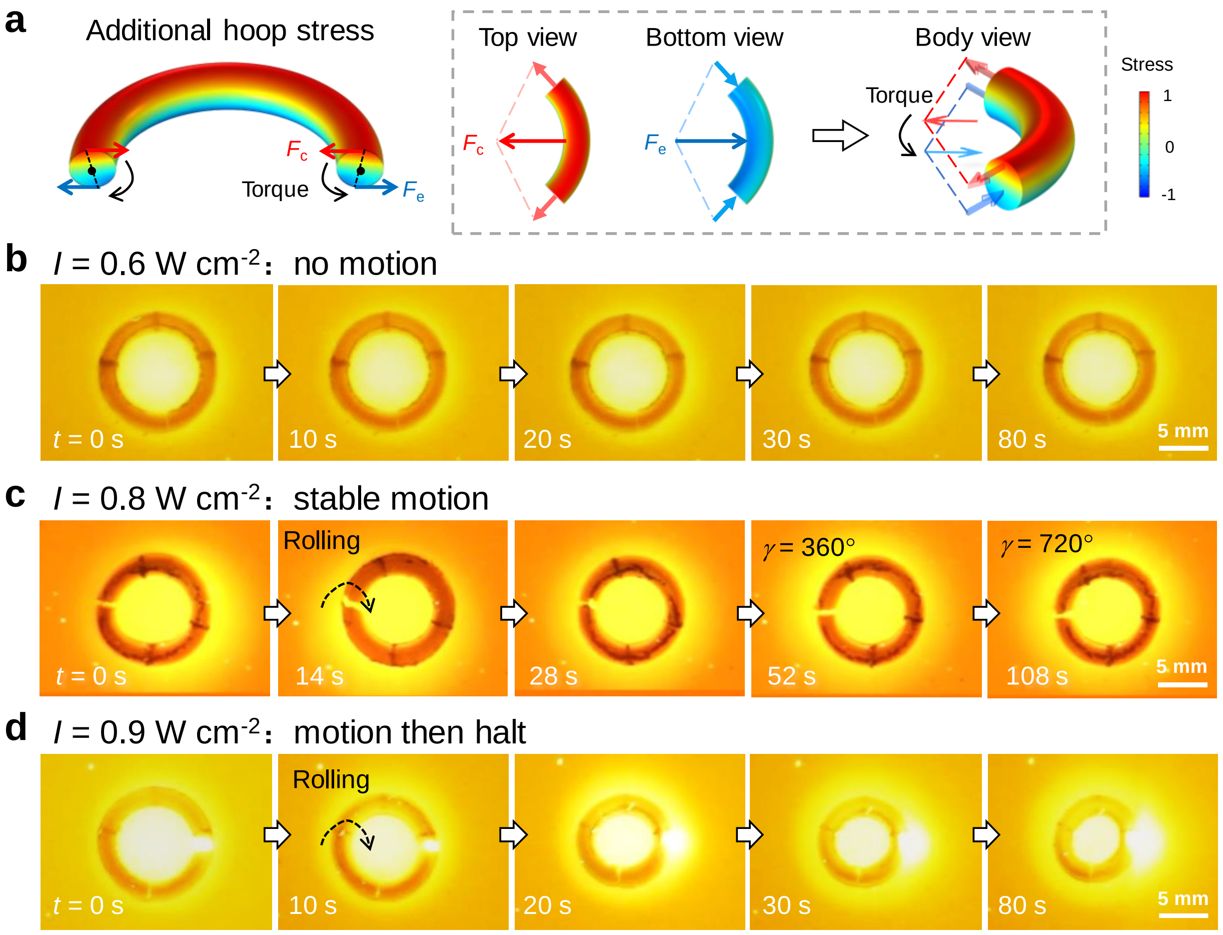

Supplementary Figure 12. Continuous rolling motion of a T_0 torus knotbot under uniform light irradiation. a Simulation result of the additional hoop stress within a T_0 torus knotbot under uniform light irradiation. This hoop stress is the driving force for rolling motion. F_c and F_e represents the resultant concentric force and eccentric force, respectively. **b-d** Photos showing the continuous motions of a T_0 knotbot under uniform light irradiation with different power intensity. When the power intensity is relatively low (0.6 Wcm^{-2}), the hoop stress induced by temperature gradient is insufficient to activate the motion of T_0 torus. When the power intensity increases to 0.8 W cm^{-2}, the T_0 knotbot exhibits continuous rolling motion at a speed of 6.8 ° s^{-1}. With further increase in power intensity to 0.9 W cm^{-2}, the T_0 knotbot rolls inward in the first 30 s and then ceases gradually due to relatively slow heat dissipation and disappearance of temperature gradient within the gel torus.

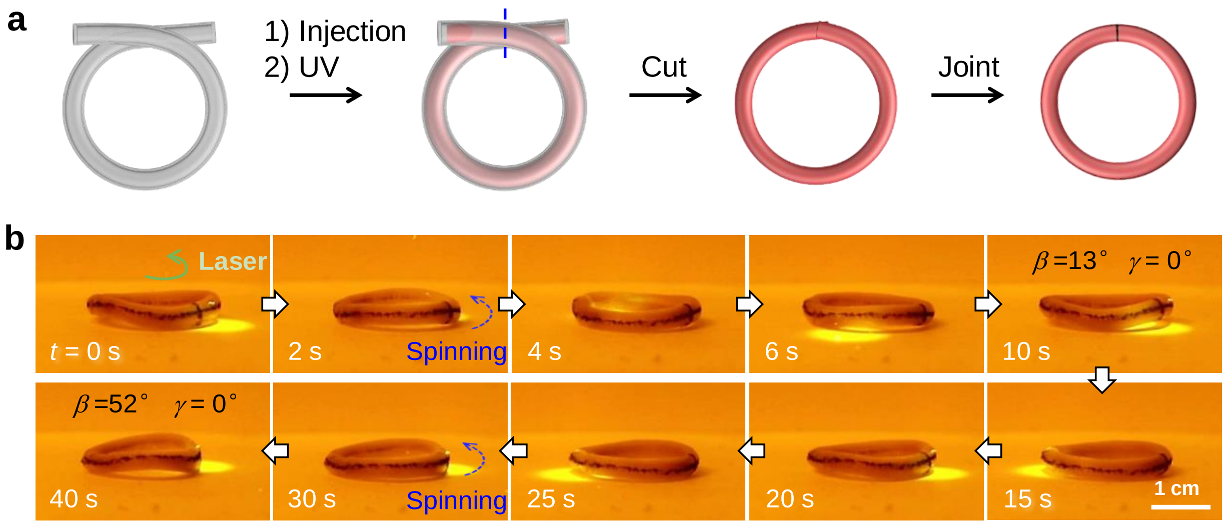

Supplementary Figure 13. Fabrication of a gel torus without topological prestrain and its motion under scanning light irradiation. a Schematic for the preparation of a gel torus without topological prestrain by using a tube as the mold. **b** Photos showing the motion under scanning light irradiation at room temperature. The molded gel torus without prestrain exhibits spinning motion induced by traveling bending deformation, but no rolling motion as it fails to overcome the energy barrier. The directly molded torus with intrinsic curvature lacks the self-similarity against rolling, and the induced bending energy strongly resists the rolling motion. Power intensity of the scanning laser is 1.2 W cm^{-2}.

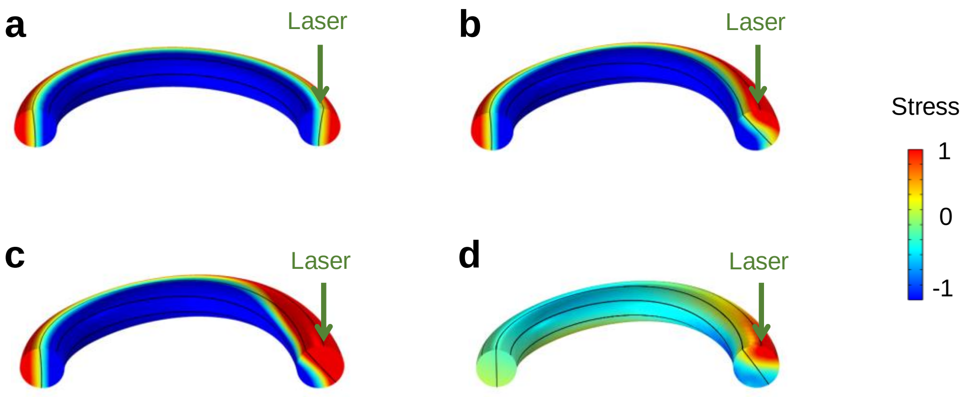

Supplementary Figure 14. Hoop stress in a knotbot subjected to a light spot irradiation. a-c Normalized hoop stress in a T_0 knotbot before (a), at a steady state after continuous light irradiation (b), and after the motion is stalled during irradiation (c), with a light spot atop. **d** Differential hoop stress between (b) and (c), showing the contribution from light irradiation.

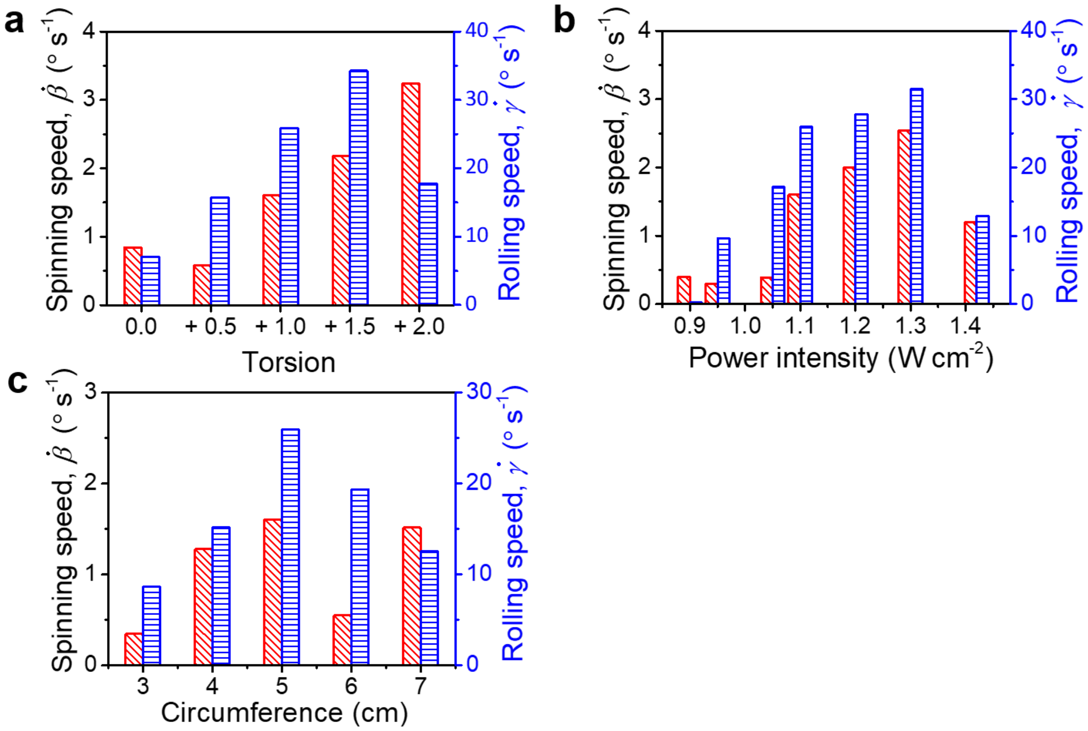

Supplementary Figure 15. Effects of torsional strain, power intensity, and size on rolling and spinning motions of torus knotbots. a Effect of torsional prestrain introduced at preparation on the spinning and rolling speeds under scanning light irradiation at room temperature. Power intensity of the scanning laser is 1.2 W cm^{-2}. **b** Effect of power intensity on the spinning and rolling velocities of a T_{+1} knotbot. **c** Influence of the overall size on the initial spinning and rolling velocities of a T_{+1} knotbot. Scanning speed of the laser spot is constant at 5 mm s^{-1}. A knotbot of smaller size (circumference < 4 cm) has a shorter time window for cooling, and thus gradually contracts and slows down the spinning and rolling motions. A knotbot of larger size (circumference > 5 cm) has a smaller curvature and thus smaller resultant rolling moment from the hoop stress, and the smaller active region relative to the overall size also slows down the rolling and spinning motions.

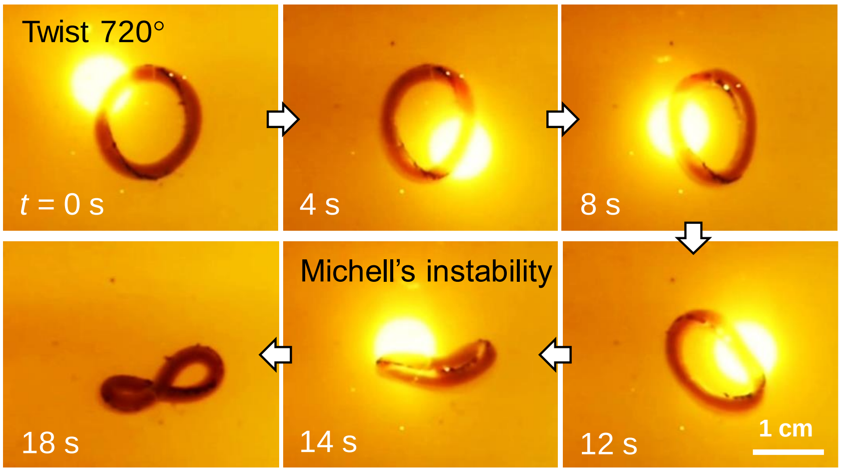

Supplementary Figure 16. Michell's instability of a T_{+2} knotbot under anticlockwise scanning light irradiation. The T_{+2} torus knotbot with a large torsional prestrain maintains the saddle-like configuration at room temperature. Under the scanning light irradiation, which generates temperature gradient and changes the distribution of internal stress within the gel torus, the T_{+2} knotbot loses stability and deforms into a figure-of-eight loop to reduce elastic energy. Power intensity, 1.2 W cm^{-2}; scanning speed, 5 mm s^{-1}.

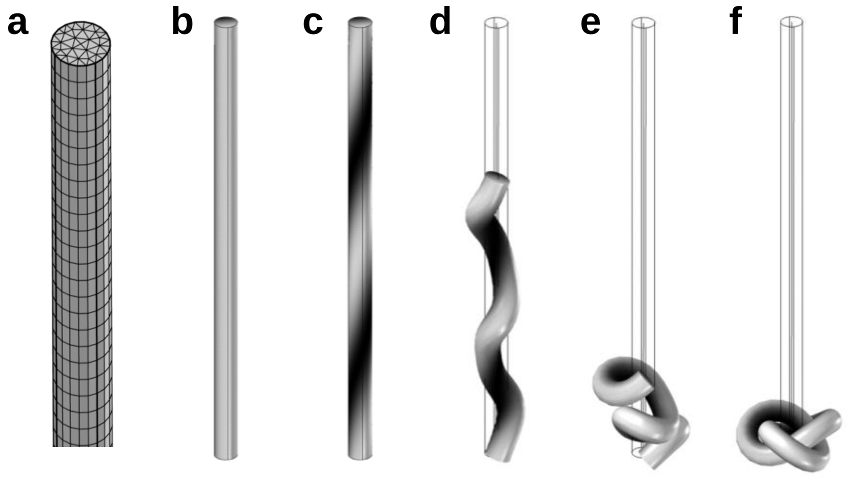

Supplementary Figure 17. Preparation of the numerical model of a knotbot. a Mesh of a straight gel noodle in the reference state. **b-f** Representative snapshots of the steps to connect a strand into a knotbot in finite element calculation: reference state (b), twisted state (c), and compressive state without rolling (d-f).

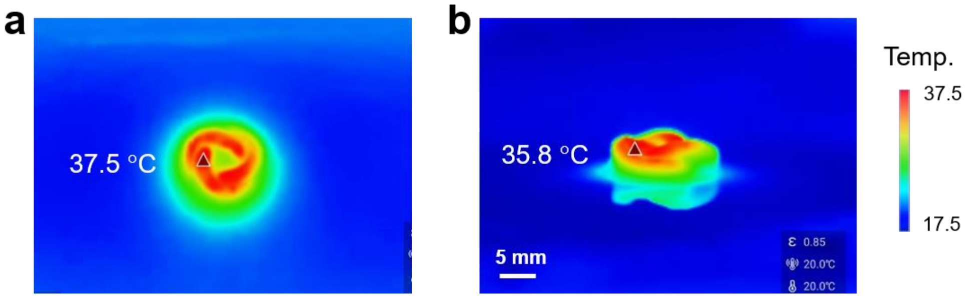

Supplementary Figure 18. Infrared photos showing the temperature distribution of a trefoil knotbot under uniform light irradiation from the top. The photos are taken from top view (a) and side view (b) of a trefoil knotbot exposed to light irradiation for 3 s at room temperature. The surficial temperature of the knobot quickly increases to a value above the LCST of PNIPAm because of the high photothermal efficiency of AuNPs. The result indicates a temperature gradient in the vertical direction of the knotbot due to the self-shadowing effect.

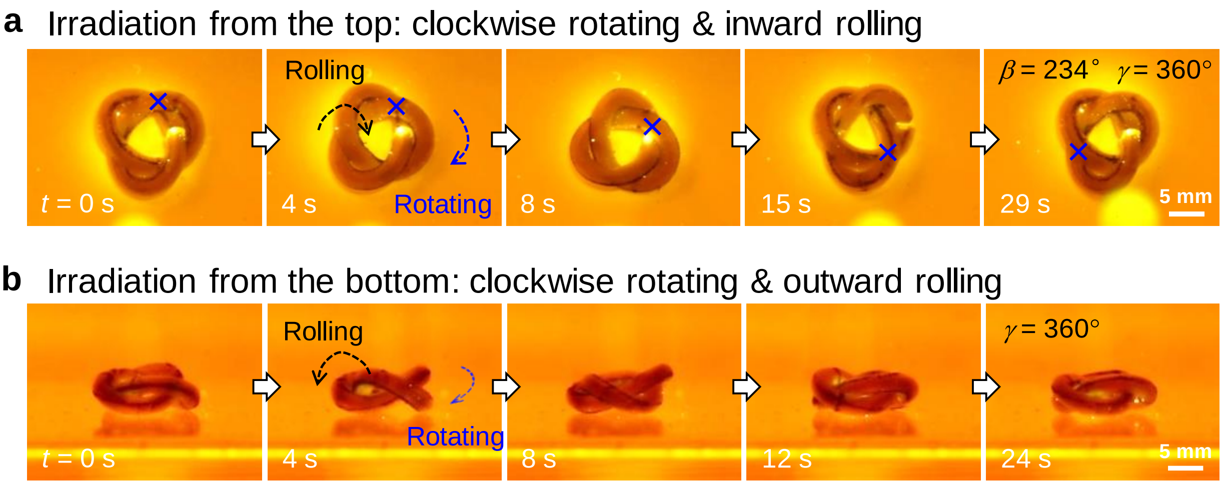

Supplementary Figure 19. Influences of the chirality and the direction of light irradiation on the motion direction of trefoil knotbots. a Clockwise braid rotation and inward rolling of left-handed trefoil knotbot under uniform light irradiation from the top. **b** Clockwise braid rotation and outward rolling of right-handed trefoil knotbot under uniform light irradiation from the bottom. Power intensity, 0.8 W cm^{-2}.

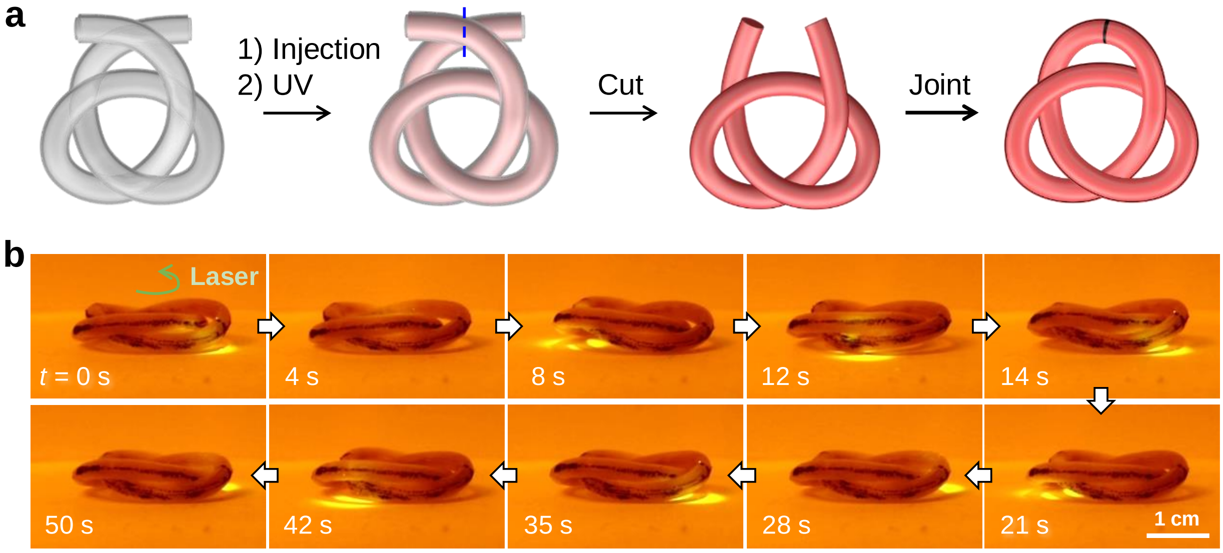

Supplementary Figure 20. Fabrication of a trefoil gel without topological prestrain and its deformation under light scanning irradiation. a Schematic for the preparation of a trefoil gel without topological prestrain by using a knotted soft tube as the mold. **b** Photos showing the deformation of the resultant trefoil gel under anticlockwise scanning light at room temperature. Without prestrain, the molded trefoil gel fails to overcome the energy barrier against rolling. Power intensity, 1.2 W cm^{-2}; scanning speed, 5 mm s^{-1}.

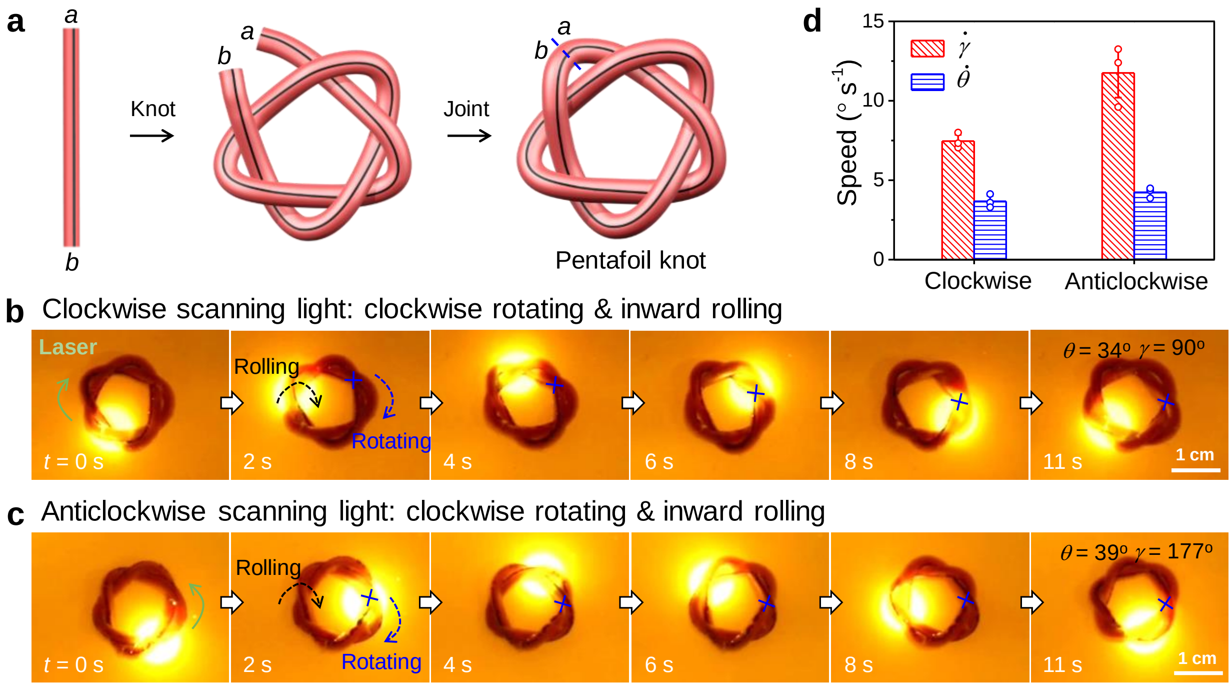

Supplementary Figure 21. Rolling and braid rotation of a left-handed pentafoil knotbot upon scanning light irradiation. a Schematic for the fabrication of the left-handed pentafoil knotbot. **b**, **c** The rolling and braid rotation of the pentafiol knotbot under clockwise (b) and anticlockwise (c) scanning light irradiation at room temperature. The circumference of the pentafoil knotbot is 12 cm. **d** Braid rotation speed $\dot{\theta}$ and rolling speed $\dot{\gamma}$ of the pentafoil knotbot under clockwise and anticlockwise scanning light. Power intensity, 1.2 W cm^{-2}; scanning speed, 5 mm s^{-1}. Data points are represented as mean ± s.d. ($n \geq 3$).

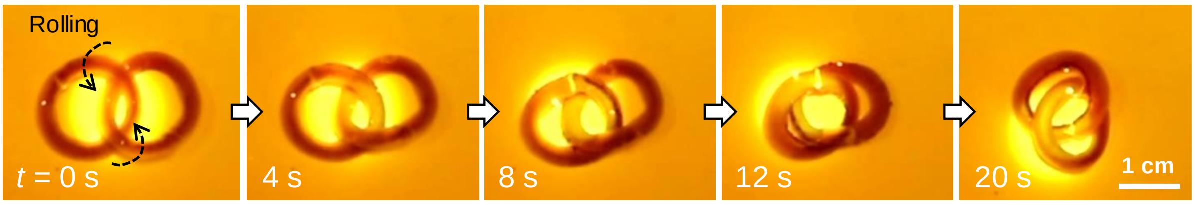

Supplementary Figure 22. Light-irradiation-induced motion of a Hopf-link knotbot. Upon light irradiation on the crossings at room temperature, the interlocked tori of Hopf link exhibit rolling motions, which drive them closer. The crossings of Hopf link facilitate self-shadowing effect to regulate the local deformation and result in translational displacement. The circumference of each ring is 3 cm. Power intensity, 1.2 W cm^{-2}.

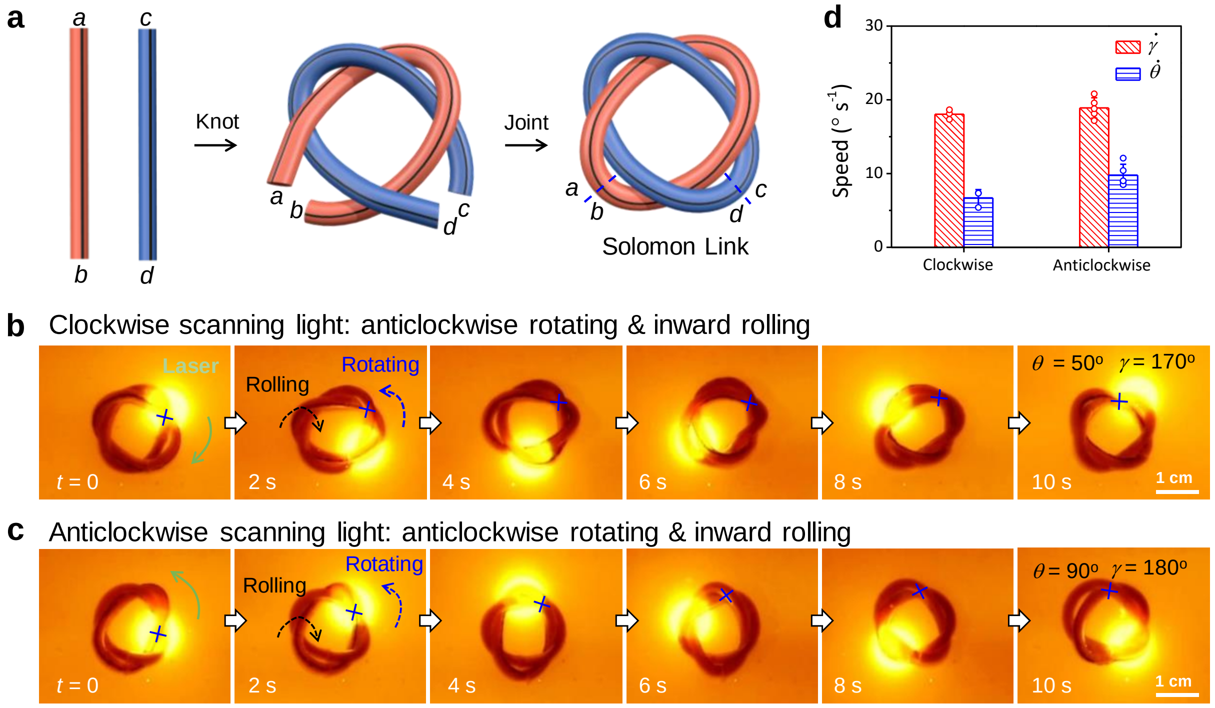

Supplementary Figure 23. Braid rotation and rolling motion of a Solomon-link knotbot upon scanning light irradiation. a Schematic for the fabrication of a right-handed Solomon-link knotbot. **b**, **c** Photos showing clockwise braid rotation and inward rolling of the knotbot under scanning light irradiation at room temperature. **d** Braid rotation speed $\dot{\theta}$ and rolling speed $\dot{\gamma}$ of the knotbot under clockwise and anticlockwise scanning light. The circumference of each strand is 6 cm. Power intensity, 1.2 W cm^{-2}; scanning speed, 5 mm s^{-1}. Data points are represented as mean ± s.d. ($n \geq 3$).

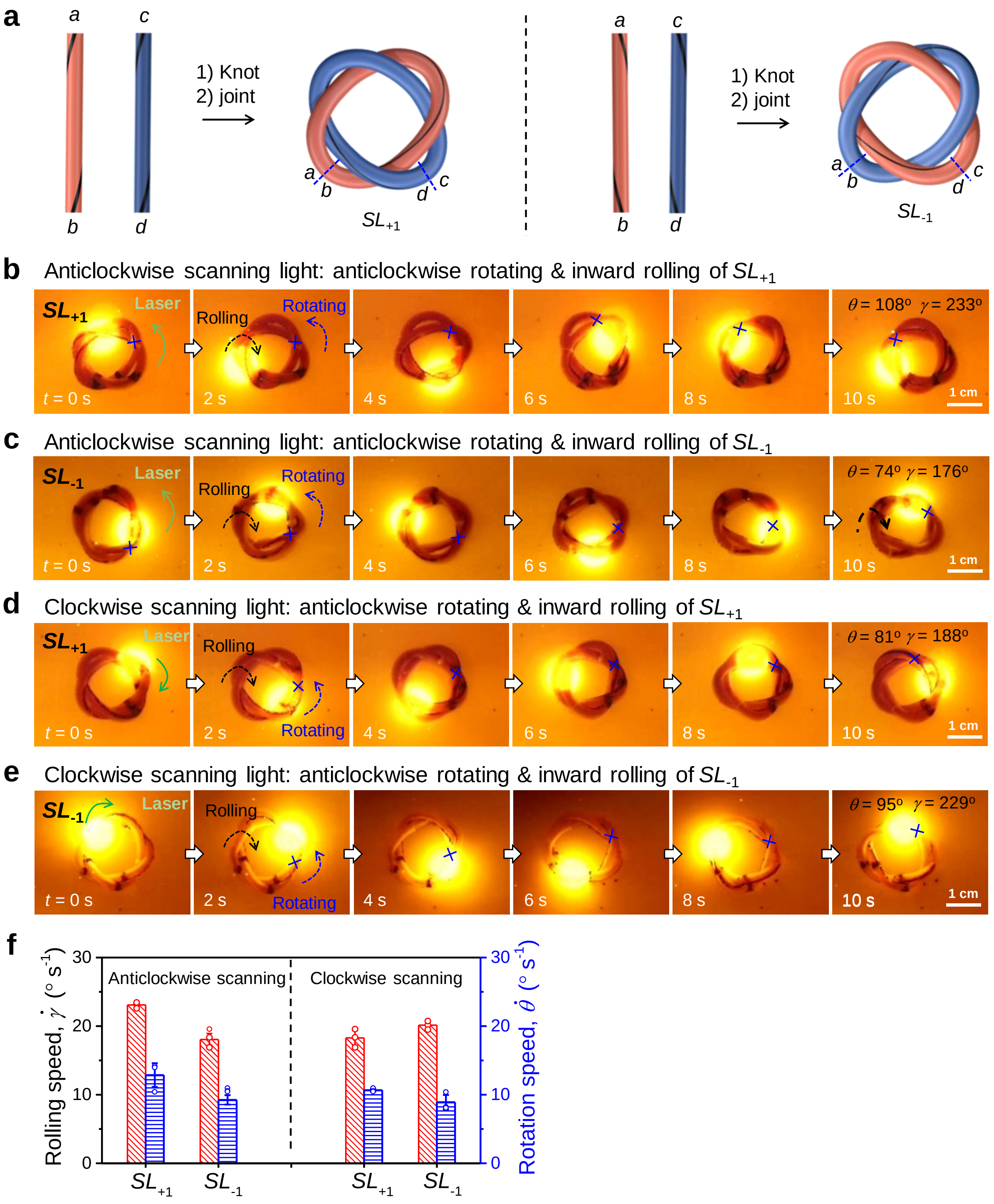

Supplementary Figure 24. Braid rotation and rolling motion of Solomon-link knotbots with torsional prestrain upon scanning light irradiation. a Schematic for the fabrication of right-handed Solomon-link knotbots with built-in torsional strain. **b**, **c** Braid rotation and rolling motion of SL_{+1} (b) and SL_{-1} (c) knotbots under anticlockwise scanning light at room temperature. **d**, **e** Braid rotation and rolling motion of SL_{+1} (d) and SL_{-1} (e) under clockwise scanning light at room temperature. **f** Braid rotation speed $\dot{\theta}$ and rolling speed $\dot{\gamma}$ of SL_{+1} and SL_{-1} knotbots under clockwise and anticlockwise scanning light irradiation. The circumference of each torus in the Solomon link is 6 cm. Power intensity, 1.2 W cm^{-2}; scanning speed, 5 mm s^{-1}. Data points are represented as mean ± s.d. ($n \geq 3$).

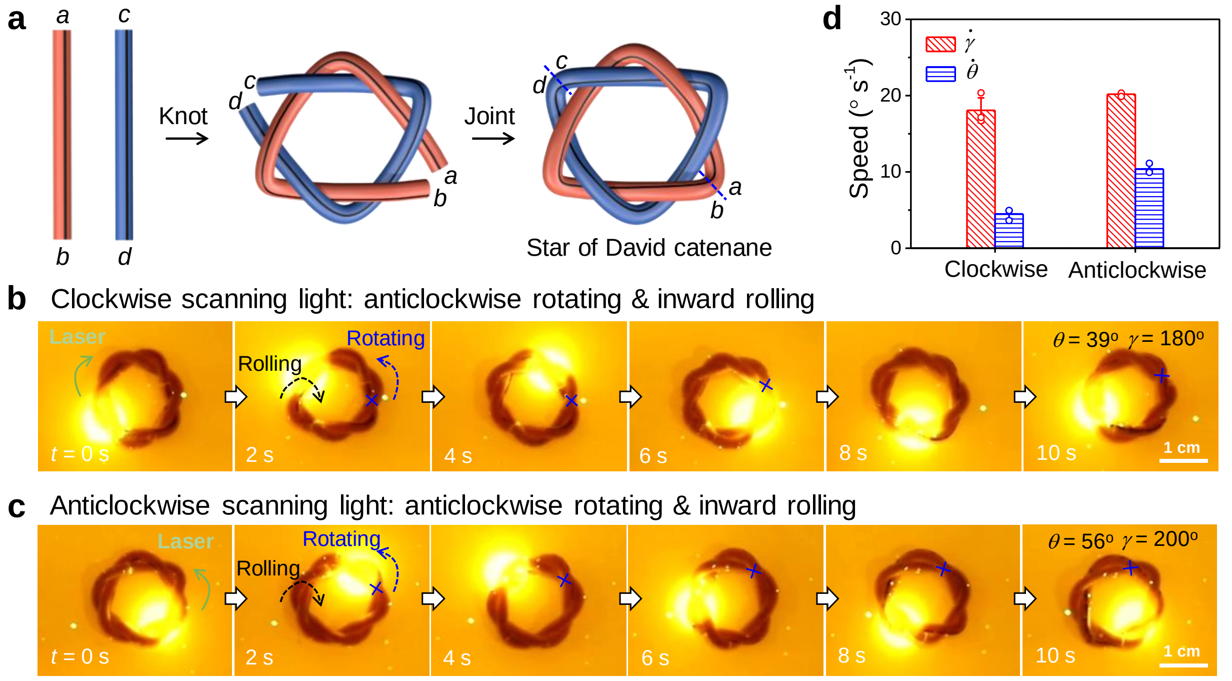

Supplementary Figure 25. Braid rotation and rolling motion of a right-handed Star-of-David link knotbot under scanning light irradiation. a Schematic for the fabrication of right-handed Star-of-David link knotbot. **b**, **c** Braid rotation and rolling motion of a Star-of-David link knotbot under clockwise (b) and anticlockwise (c) scanning light irradiation at room temperature. **d** Braid rotation speed $\dot{\theta}$ and rolling speed $\dot{\gamma}$ of the Star-of-David link knotbot under clockwise and anticlockwise scanning light irradiation. The circumference of each torus in the Star-of-David link knotbot is 8 cm. Power intensity, 1.2 W cm^{-2}; scanning speed, 5 mm s^{-1}. Data points are represented as mean ± s.d. ($n \geq 3$).

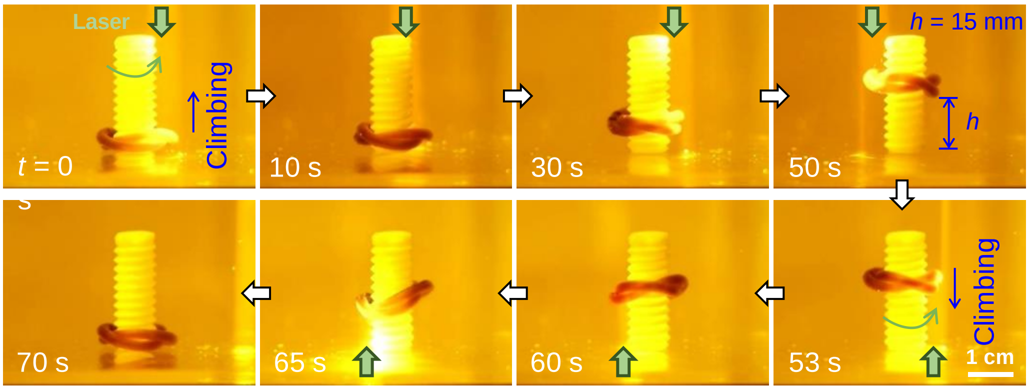

Supplementary Figure 26. Climbing motion of a trefoil knotbot along a vertical threaded rod upon scanning light irradiation at room temperature. The climbing motion arises from the inward rolling of the trefoil knotbot under light irradiation, and the climbing direction depends on the direction of light irradiation. Power intensity, 1.2 W cm^{-2}. The diameter of the threaded rod is 10 mm, which is comparable to the size of trefoil knotbot.

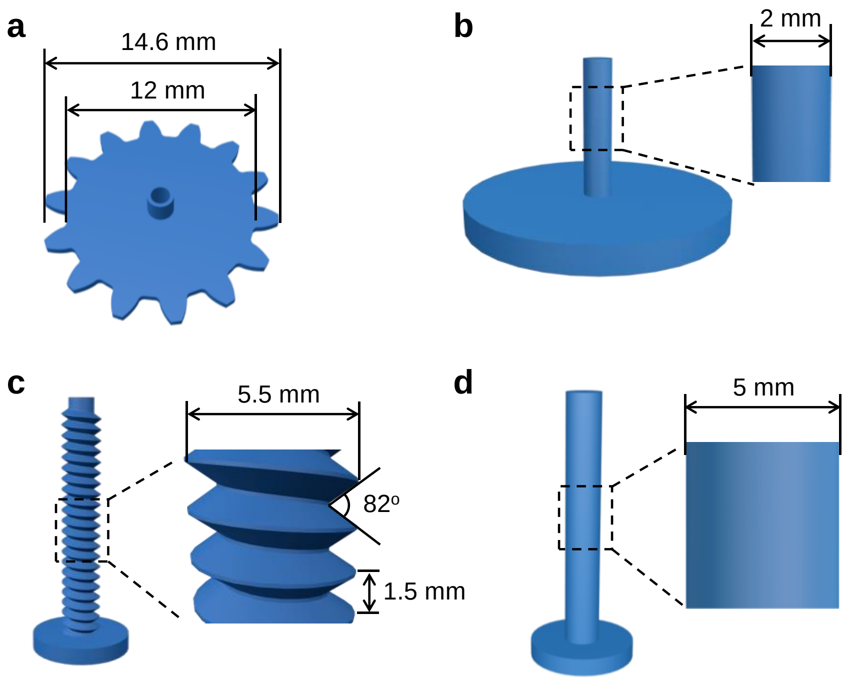

Supplementary Figure 27. Dimensions of the elements used in Figure 5. a, **b** Dimensions of the gear (a) and the base with a rod (b) used in Fig. 5a. **c**, **d** Dimensions of threaded (c) and unthreaded (d) rods used in Fig. 5b and Fig. 5c, respectively.

Supplementary Table

Supplementary Table 1. SAXS measurement of the anisotropic nanocomposite hydrogel. *d*-spacing between the cofacially-aligned NSs within the hydrogel calculated from the scattering peaks in Supplementary Fig. 6. (*hkl*) are the Miller indices.

**(*hkl*)**	***q*** (nm^{-1})	***d*-spacing** (nm)
100	0.171	36.7
200	0.338	18.6
300	0.529	11.9
400	0.706	8.9
500	0.878	7.2

Supplementary References

1. Majima, T., Schnabel, W. & Weber, W. Phenyl-2,4,6-trimethylbenzoylphosphinates as water-soluble photoinitiators - generation and reactivity of O=P(C6h5)(O-) radical-anions. *Macromol. Chem. Phys.* **192**, 2307-2315 (1991).
2. Rosenfeldt, S. *et al.* In-depth insights into the key steps of delamination of charged 2D nanomaterials. *Langmuir* **32**, 10582-10588 (2016).
3. Daniel, A. K. *et al.* In-plane modulus of singular 2:1 clay lamellae applying a simple wrinkling technique. *ACS Appl. Mater. Interfaces* **5**, 5851-5855 (2013).
4. Frens, G. Controlled nucleation for the regulation of the particle size in monodisperse gold suspensions. *Nat. Phys. Sci.* **241**, 20-22 (1973).
5. Hong, S. H., Shen, T. Z. & Song, J. K. Flow-induced alignment of disk-like graphene oxide particles in isotropic and biphasic colloids. *Mol. Cryst. Liq. Cryst.* **610**, 68-76 (2015).
6. Alizadehgiashi, M. *et al.* Shear-induced alignment of anisotropic nanoparticles in a single- droplet oscillatory microfluidic platform. *Langmuir* **34**, 322-330 (2018).
