# Supplementary material for: Animating hydrogel knotbots with topology-invoked self-regulation
Source: Nat Commun. 2024 Jan 5;15:300. doi: 10.1038/s41467-023-44608-x (PMC10770334; doi:10.1038/s41467-023-44608-x)
Supplement: Supplementary file 3 — Description of Additional Supplementary Files [file 41467_2023_44608_MOESM3_ESM.pdf]

Description of Additional Supplementary Files

File name: Supplementary Movie 1.
Description: Reversible bending and unbending of anisotropic cylindrical hydrogel under intermittent irradiation of a light spot. Diameter of gel, 2.0 mm; light wavelength, 520 nm; power intensity, 0.8 W cm^{-2}. Movie speed, 5×.

File name: Supplementary Movie 2.
Description: Continuous rolling of a T_0 knotbot under uniform light irradiation and corresponding finite-element simulation. Circumference of torus gel, 4 cm; light wavelength, 520 nm; power intensity, 0.8 W cm^{-2}. Movie speed, 5×.

File name: Supplementary Movie 3.
Description: Continuous rolling and spinning of T_0 and T_{+1} knotbots under scanning light irradiation and corresponding finite-element simulations. Circumference of torus gel, 5 cm; power intensity, 1.2 W cm^{-2}; laser diameter, 18 mm; scanning speed, 5 mm s^{-1}. Movie speed, 5×.

File name: Supplementary Movie 4.
Description: Inward rolling and braid rotation of a right-handed trefoil knotbot under uniform light irradiation from the top and corresponding finite-element simulation. Circumference of gel strand in the knotbot, 7 cm; power intensity, 0.8 W cm^{-2}. Movie speed, 10×.

File name: Supplementary Movie 5.
Description: Inward rolling and braid rotation of right-handed trefoil knotbot under anticlockwise and clockwise scanning light irradiation and corresponding finite-element simulations. Circumference of gel strand in the knotbot, 10 cm; laser diameter, 10 mm; power intensity, 1.2 W cm^{-2}; scanning speed, 5 mm s^{-1}. Movie speed, 5×.

File name: Supplementary Movie 6.
Description: Inward rolling and braid rotation of pentafoil, Solomon-link, and Star-of-David link knotbots under uniform light irradiation. Circumferences of gel strands in pentafoil, Solomon-link, and Star-of-David link knotbots are 8 cm, 4 cm, and 6 cm, respectively. Power intensity, 0.8 W cm^{-2}. Movie speed: pentafoil and Solomon-link knotbots, 10×; Star-of-David link knotbot, 20×.

File name: Supplementary Movie 7.
Description: Rotating a gear and climbing a vertical rod of right-handed trefoil knotbots under uniform light irradiation. Circumference of gel strand in the knotbot, 7 cm; power intensity, 0.8 W cm^{-2}. Movie speed, 10×.

File name: Supplementary Movie 8.
Description: Transportation of right-handed trefoil knotbot along a horizonal string under uniform light irradiation. Circumference of gel strand in the knotbot, 7 cm; power intensity, 0.8 W cm^{-2}. Movie speed, 5×.
